# Safe access to laparoscopic cholecystectomy in patients with previous periumbilical incsions: new approach to avoid entry related bowel injury

**DOI:** 10.1007/s00423-025-03624-9

**Published:** 2025-01-30

**Authors:** Mostafa Seif, Mohamed Mourad, Mostafa Refaie Elkeleny, Mohamed Wael

**Affiliations:** 1 Alexandria Main University Hospital, Alexandria, Egypt; 2 Alexandria University, Alexandria, Egypt

**Keywords:** Laparoscopic cholecystectomy, Adhesions, Umbilical scars

## Abstract

**Background:**

Patients with prior abdominal surgeries are at higher risk of intra-peritoneal adhesions near the trocar entry site, increasing the likelihood of organ injury during laparoscopic cholecystectomy (LC). This study evaluates a novel technique where the epigastric trocar is inserted first, after creating pneumoperitoneum, to allow safe dissection of adhesions under direct vision before placing the umbilical trocar.

**Methods:**

This prospective study included 244 patients with symptomatic uncomplicated gallstone disease and a history of previous abdominal surgeries extending to the umbilicus. Patients were randomly assigned to two groups: Group I (*n* = 98) underwent traditional umbilical trocar-first LC using the Hasson technique, while Group II (*n* = 146) received LC using the epigastric trocar-first approach. Operative time, complications, and conversion rates were analyzed.

**Results:**

There was no significant difference in the demographics between both groups. The epigastric trocar-first approach significantly reduced total operative time (41.6 ± 7.7 min vs. 46.8 ± 8.8 min, *p* = 0.031) and small bowel injury rates (*p* = 0.006). Otherwise, intraoperative complications were comparable. Conversion to open surgery was lower in Group II (2.1% vs. 8.2%, *p* = 0.012). Postoperative pain at 6 h was significantly lower in Group II (*p* = 0.001).

**Conclusions:**

The epigastric trocar-first approach, offers a safer alternative for patients with prior abdominal surgeries when undergoing LC. This approach is safe; minimizes bowel injury risk, reduces conversion rates, and enhances patient recovery. This approach may also be beneficial in other laparoscopic procedures requiring safe entry in patients with prior abdominal surgeries. Further studies are recommended to validate its broader clinical application.

## Introduction

Laparoscopic cholecystectomy (LC) is the standard treatment for gallstone disease; however, entry-related complications remain a concern, particularly in patients with prior abdominal surgeries. In this category of patients, adhesions near the umbilicus increase the risk of abdominal visceral injury during trocar placement; the most significant are the intestinal and vascular injuries. These injuries are specific to laparoscopy and are rarely seen during open surgery [1, 2]. 

Various laparoscopic entry techniques are available including: the classic Veress needle method for pneumoperitoneum, the open (Hasson) technique and different techniques of direct trocar insertion. The initial access is typically achieved in these techniques without direct visualization, i.e.: in a blind fashion. Reviewing the literature, there is no single technique or instrument that has been proven to completely prevent injuries associated with laparoscopic entry [3]. 

The umbilicus is conventionally used for trocar insertion due to its central position and low vascularity [4, 5]. However, in patients with previous umbilical or midline incisions, intraperitoneal adhesions can increase the risk of visceral injury during blind trocar placement. This scenario raises the important consideration of whether utilizing different laparoscopic entry sites could enhance safety for this specific group of patients instead of the traditional laparoscopic entry near the umbilicus [6]. 

Given the challenges of umbilical entry in patients with prior abdominal surgeries, we introduced a new novel epigastric trocar-first technique to enhance LC safety, where pneumoperitoneum is first created via Veress needle insertion at the Palmars point, followed by epigastric trocar placement under direct vision. This approach aims to enhance safety by allowing dissection of adhesions before umbilical trocar insertion, reducing the risk of injury. We compared its outcomes with the traditional umbilical trocar-first technique using the classic Hasson open technique for entry aiming at assessment of the efficacy of our novel approach in patients with prior abdominal surgeries. The results were analyzed in this study.

## Materials and methods

### Patient selection

This one-year prospective study was carried out on 244 patients with symptomatic uncomplicated gallstone disease with a history of umbilical or abdominal surgeries with scars extending to the umbilicus. The research took place at the Alexandria Main University Hospital, a 1000-bed teaching facility affiliated with the Faculty of Medicine at the University of Alexandria, along with several non-governmental hospitals. Prior approval from the Institutional Ethics Committee was taken before commencement of the study.

**Patients having one or more of the following conditions were excluded from the study: a)** complicated cholecystitis e.g. acute cholecystitis or empyema of the gall bladder. **B)** Suspected gallbladder malignancy (based on ultrasound/CT findings). C) Associated choledocholithiasis requiring common bile duct exploration. **D)** Associated conditions affecting surgical safety (e.g., splenomegaly, gastric outlet obstruction, large paraumbilical hernias). **E)** Previous open upper abdominal surgery with suspected difficult epigastric trocar insertion. F) Any contraindication to laparoscopy.

Following a comprehensive preoperative assessment, Patients were randomly assigned via the closed-envelope method into two groups: **Group I** (*n* = 98): Underwent traditional **umbilical trocar-first LC** using the Hasson open technique. **Group II** (*n* = 146): Underwent **epigastric trocar-first LC**, utilizing the novel technique. Informed consent was obtained from all participants after the benefits and risks were explained.

### Surgical management (anesthesia/surgical principles)

All patients received perioperative prophylactic antibiotics together with antiemetic at induction as well as general anesthesia with endotracheal intubation.

**Group I**: ***Umbilical trocar entry first laparoscopic cholecystectomy*group (***n*** = 98)**:

Under general anesthesia, the patient was placed in a supine position. A 2-cm curved incision was made at the umbilicus, followed by layer-by-layer dissection down to the rectus fascia. In cases where the umbilicus has been previously excised; the incision is positioned at the midpoint between the xiphisternum and the symphysis pubis. A purse-string suture was applied to maintain pneumoperitoneum after trocar insertion. The Hasson open technique was used to insert a 12-mm blunt trocar for camera access, and the purse string suture is secured once, under tension, to maintain an airtight seal [6, 7]. 

**Fig. 1:**
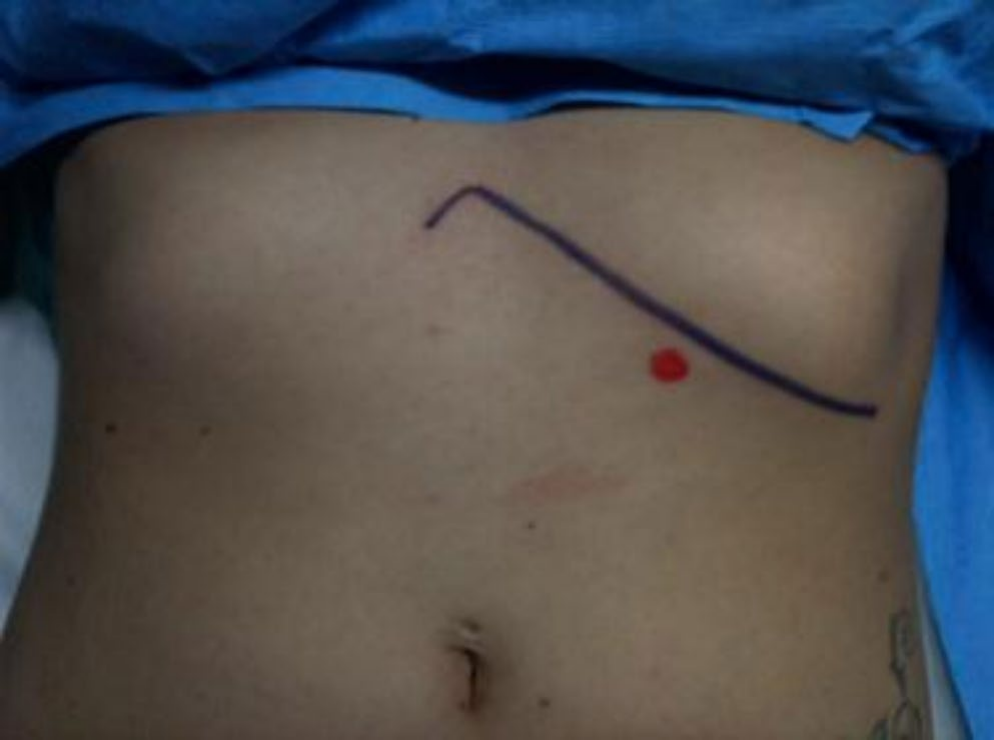
Position of Palmars point [9]

**Fig. 2:**
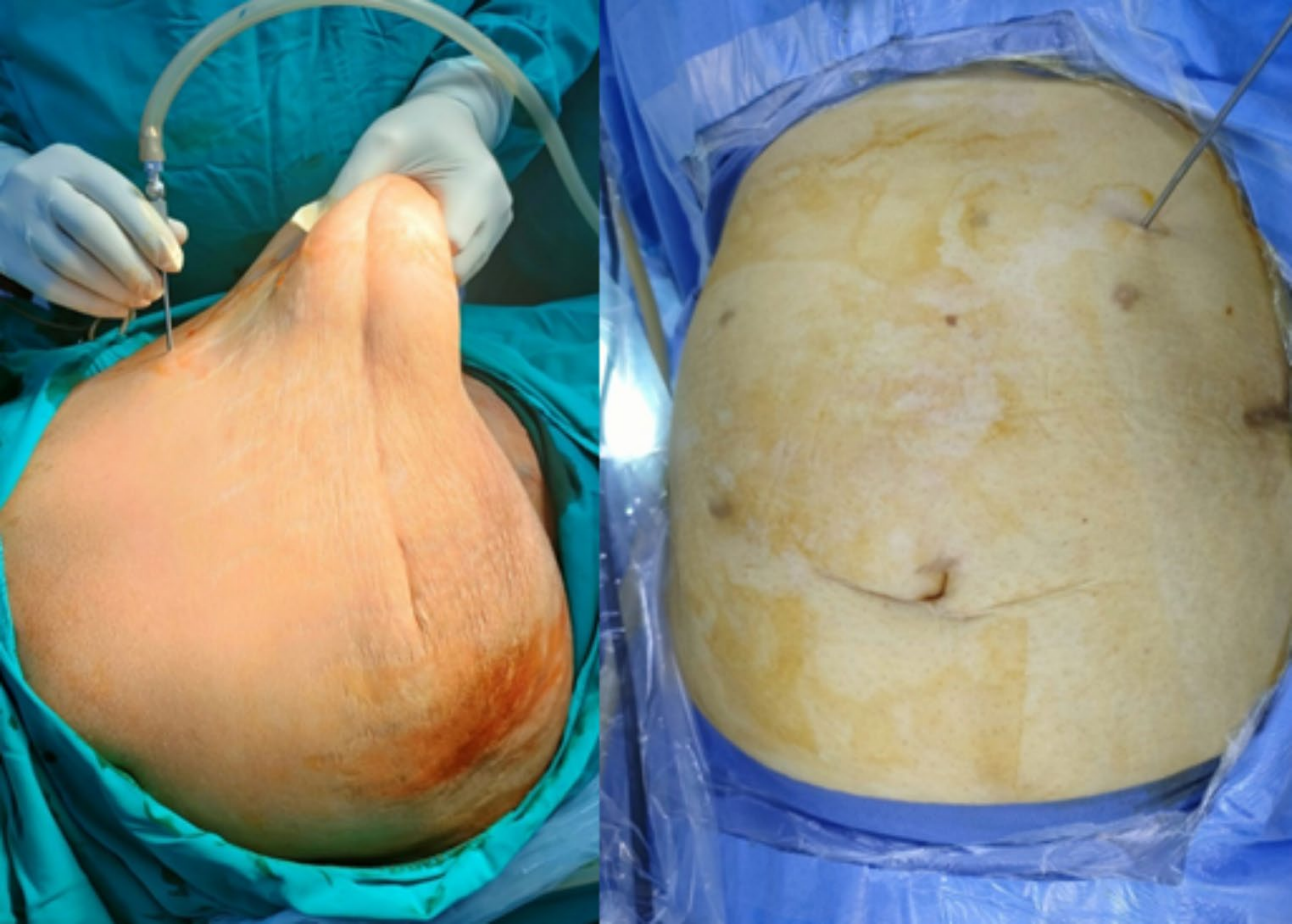
Veress needle is inserted through a skin puncture in the palmars point (photo from our cases)

**Fig. 3:**
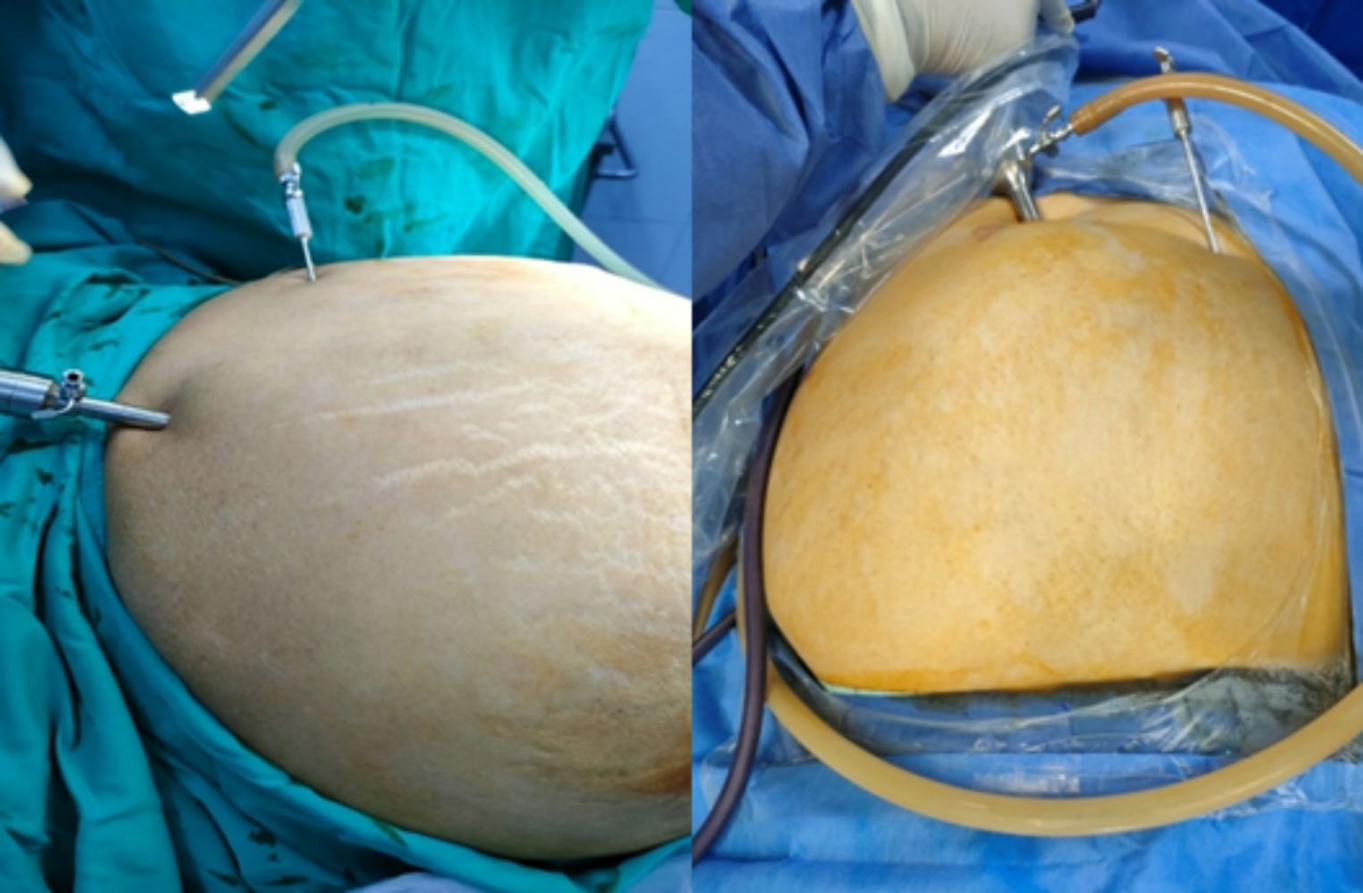
After creation of pneumoperitoneum, insertion of the epigastric trocar first

**Fig. 4:**
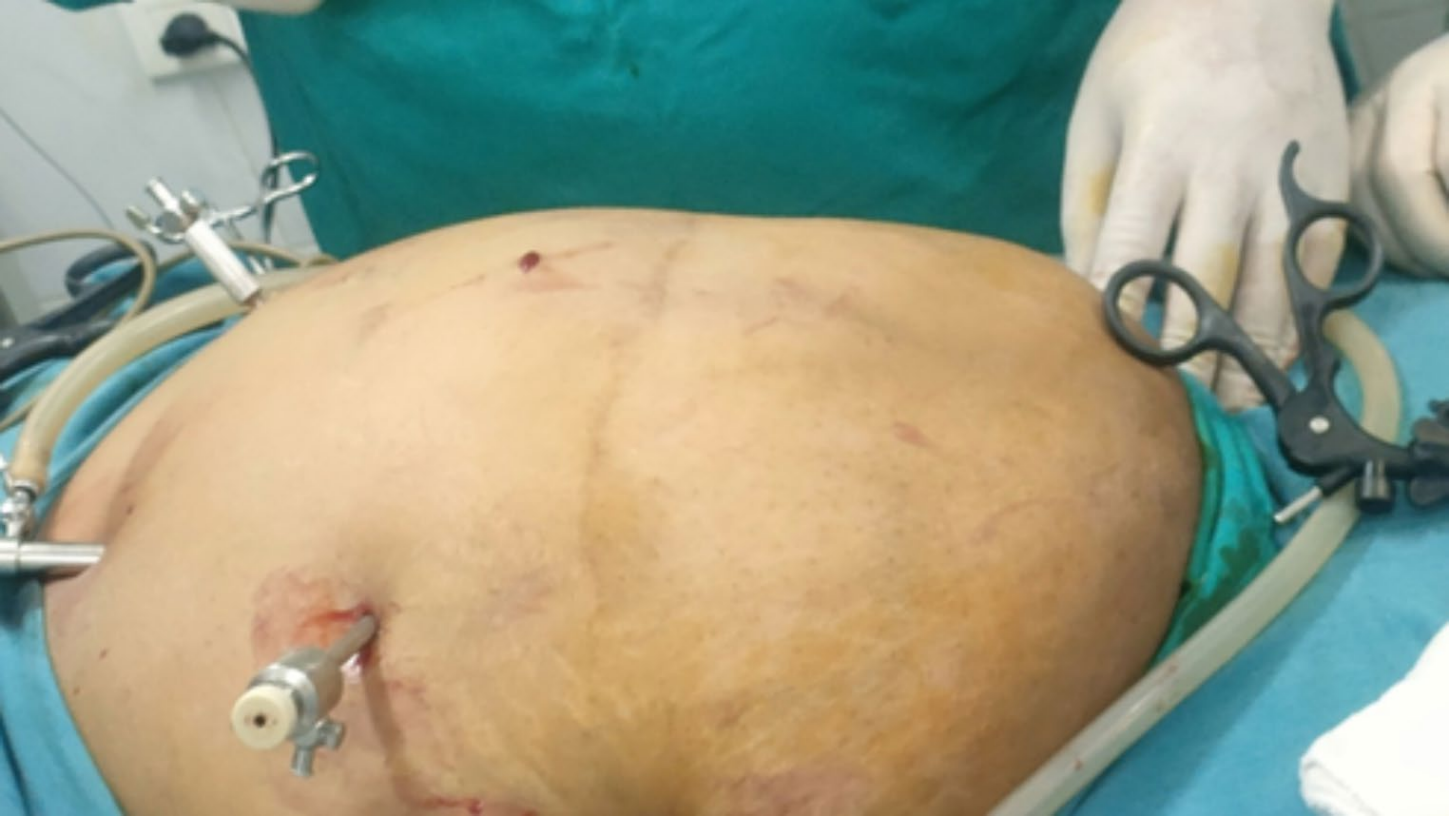
Insertion of mid-clavicular and anterior axillary trocars in the same sites of trocars insertion as in traditional laparoscopic cholecystectomy

**Fig. 5:**
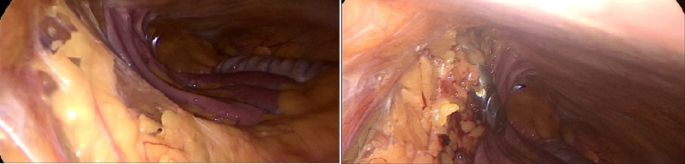
Safe entry of umbilical trocar under vision after careful dissection and lateralization of umbilicus related intra-abdominal adhesions

**Group II: Epigastric trocar entry first laparoscopic cholecystectomy (***n*** = 146)**:

In supine position, Palmars point [8], is identified as shown in Fig. 1 [9]. A puncture is made in the skin 3 cm below the left costal margin at the mid-clavicular line, after which the Veress needle is inserted (Fig. 2). The correct placement of the Veress needle within the peritoneal cavity is confirmed through several established tests; Aspiration test, Injection test, Recovery test and Saline drop test [3]. Once pneumoperitoneum is established, a 1 cm small incision is made in approximate site to the gall bladder fundus; at the junction of the lateral border of the linea semilunaris and the tip of the ninth costal cartilage. A visiport trocar is inserted under vision (Fig. 3). Camera is then introduced for formal exploration. Insertion of another mid-clavicular and anterior axillary 5 mm trocars in the same sites of trocars insertion as in the traditional laparoscopic cholecystectomy (Fig. 4) [10]. Then, adherent structures in the umbilical region are identified from within the abdominal cavity. Dissection of intra-peritoneal umbilical adhesions is then performed under direct vision. After achieving a safe access for entry, the umbilical port is then inserted, distanced from any adherent structures to the abdominal wall. (Fig. 5)

In both techniques, the table is then adjusted to place the patient in a reverse Trendlenberg position. The gallbladder was dissected using a standard four-port technique, ensuring critical view of safety before division of the cystic duct and artery [11]. Hemostasis was then ensured. Operative details were recorded with special attention to operative time and complications.

### Outcome measurements and definitions

- Preoperative data collected were demographics, BMI and the indications of laparoscopic surgery.
- Intraoperative data collected was collected. These included:

1- Duration of the procedure: The *total operative time* was defined as “the time elapsing between the first skin incision (the periumbilical incision in the *Umbilical trocar first LC*, the skin puncture for veress needle insertion in the *Epigastric trocar entry first LC*) and the last skin stitch”. *The entry time* was defined as “the time taken to a) *Umbilical trocar entry first LC*: the time to create the umbilical incision, till complete abdominal insufflation, b) *Epigastric trocar entry first LC*: the time needed for the veress needle insertion in the palmars point followed by trocar insertion in the right upper quadrant till complete abdominal insufflation. ‘’ The *Dissection time* was defined as “time from start of Calot’s triangle dissection to gallbladder extraction”.

2- the need for conversion to open or conventional cholecystectomy surgery

3- Intra-operative complications

c) Postoperative complications.

### Follow-up

At the end of the first and second postoperative weeks, patients underwent physical assessment and abdominal ultrasonography if clinically indicated. Blood samples were taken when indicated; if not, patients were reassured.

### Statistical analysis

Statistical analysis was performed using IBM SPSS Statistics for Windows, version 25.0. Categorical variables were compared with the chi-square test or Fisher’s exact test. Continues variables were compared using Student’s t test or Mann-Whitney U test. Data presented as means ± standard deviation if normally distributed or median and interquartile range if not normally distributed. We test normality using Shapiro-wilk test. Univariate analysis was performed to identify possible risk factors of CR-POPF, and logistic regression was performed to determine independent risk factors for CR-POPF used. P-value of ≤ 0.05 were regarded as significant.

## Results

A total of 244 consecutive patients, with history of umbilical or abdominal surgeries with scars extending to the umbilicus, indicated for laparoscopic cholecystectomy were eligible for the study.

- **Demographics**,** comorbidities and indications for LC of the included patients**: Both groups had comparable baseline characteristics, with no significant differences in age, sex, BMI, comorbidities, or prior surgical history (*p* > 0.05). The primary indication for LC was symptomatic gallstone disease, present in 94% of Group I and 97% of Group II (*p* = 0.191). The demographics of the included patients, the indications for undergoing LC and details regarding comorbidities and prior surgical history are shown in Tables 1 and 2.
- **Operative details: Total operative time** was significantly lower in Group II (41.6 ± 7.7 min) compared to Group I (46.8 ± 8.8 min, *p* = 0.031). This discrepancy is primarily due to the significantly shorter entry time in the epigastric trocar first group compared to the umbilical trocar first group (4.5 ± 0.5 min vs. 8.6 ± 1.9 min, *p* = 0.001).The dissection time was slightly lower in the epigastric trocar first group (36.5 ± 8.9 min vs. 38.2 ± 8.5 min, *p* = 0.07), but the difference was not statistically significant. The detailed operative time is shown in Table 3.

**Intraoperative complications** (Table 4):

- Biliary complications: The incidence of common bile duct (CBD) injury was comparable between both groups, with two cases (2.0%) in Group I and three cases (2.1%) in Group II (*p* = 0.993). Gallbladder perforation during dissection was observed in six patients (6.1%) in Group I and nine patients (6.2%) in Group II, showing no significant difference between the groups (*p* = 0.989).
- Bowel injury: Small bowel injuries occurred significantly more in Group I compared to Group II (6.1% vs. 0.7%, *p* = 0.006). In Group I, six patients sustained small bowel injuries, including four cases of serosal tears repaired with vicryl sutures and two full-thickness injuries requiring layered closure. Conversely, in Group II, only one patient experienced a small bowel injury while attempting to dissect an adherent small bowel loop, which was successfully repaired. Colonic injuries were rare and comparable between the groups, with one patient (1.0%) in Group I sustaining a full-thickness sigmoid colon injury, and one patient (0.7%) in Group II experiencing a puncture to the transverse colon, both of which were successfully repaired (*p* = 0.775). This finding suggest that performing adhesolysis under direct vision before umbilical trocar placement minimizes the risk of inadvertent bowel injury.
- Mesenteric injuries: Four patients subjected mesenteric injuries, all in the umbilical trocar first group, that were treated with direct repair and hemostatic control. (*p* = 0.065)
- Vascular injuries: One patient in group I suffered a significant parietal abdominal wall bleeding that was controlled by hemostatic suturing. There was no significant difference between both groups.
- Liver injuries: were reported in three patients (2.1%) in Group II, all of whom experienced minor punctures from the Veress needle during pneumoperitoneum creation, which were controlled with diathermy (*p* = 0.109).
- Conversion to open approach: Conversion to open surgery was significantly higher in Group I (*n* = 8, 8.2%) vs. Group II (*n* = 3, 2.1%, *p* = 0.012). In group I, 8 patients required conversion to open laparotomy; 2 patients due to CBD injury, 2 patients due to difficult biliary anatomy, 1 patient due to colonic sigmoid injury, and 3 patients related to small bowel injuries. In contrast, 3 patients in group II were converted to laparotomy, including 2 patients for CBD injury and 1 patient for small bowel injury.

c) **Postoperative details**:

Wound infection rates were similar between groups (2% in Group I, 2.1% in Group II, *p* = 0.993); two patients in group I had wound infection in the umbilical incision while, three patients in group II had wound infection (2 patients in the umbilical incision, 1 patient in the epigastric incision) with no significant difference between both groups.

Pain at 6 h postoperatively was significantly lower in Group II (3.0 ± 0.8 vs. 4.03 ± 1.2, *p* = 0.001). Postoperative jaundice occurred in one patient per group, with residual CBD stones successfully removed via ERCP.

Additionally, subhepatic collections were reported in 1 patient (Group I) who was managed conservatively and 2 patients (Group II); one required ultrasound-guided drainage, while the other was managed conservatively (*p* > 0.05). The detailed postoperative follow up data is shown in Table 5.

**Table 1:** Comparison between the two studied groups regarding demographic data

	Group I Umbilical trocar first “*n* = 98”		Group II Epigastric trocar first “*n* = 146”		Test *P* value
**Demographic data** **Age (years)** Range Mean SD	29–58 42.3 8.4		32–61 41.9 7.9		0.932 0.352 N.S.
**Sex** Male Female		18 (18.4%) 80 (81.6%)		36 (24.7%) 110 (75.3%)	1.346 0.245 N.S.
**Body mass index (Kg/m** ^**2**^ **)** Range Mean SD	22.5–35.6 30.2 3.3		24.1–35.5 29.8 3.4		1.52 0.192 N.S.
Indication for LC		No (%)		No (%)	
**Symptomatic gall stone** **Gall bladder polyps**		92 (93.9%) 6 (6.1%)		142 (97.3%) 4 (2.7%)	1.702 0.191 N.S.

(p) p value for comparing between the two studied groups; (*) Statistically significant at *p* ≤ 0.05

**Table 2:** Comparison between the two studied groups regarding preoperative comorbidities and surgical history

	Group I Umbilical trocar first “*n* = 98”		Group II Epigastric trocar first “*n* = 146”		*P* value T test
	No	%	No	%	
Medical history					
**DM** No Yes	75 23	76.5 23.5	110 36	75.3 24.7	3.872 0.063 N.S.
**HTN** No Yes	75 23	76.5 23.5	117 29	80.1 19.9	1.366 0.242 N.S.
**Asthma** No Yes	87 11	88.8 11.2	126 20	86.3 13.7	2.506 0.113 N.S.
**Hyperthroidism** No Yes	84 14	85.7 14.3	125 21	85.6 14.4	3.981 0.045 N.S.
**HCV** No Yes	90 8	91.8 8.2	131 15	89.7 10.3	0.306 0.580 N.S.
**Chronic renal disease** No Yes	90 8	91.8 8.2	137 9	93.8 6.2	0.361 0.547 N.S.
**Surgical history**					
**Surgery near the umbilicus** Paraumbilical Hernia repair Abdominoplasty Laparoscopic pancreatic surgery previous Laparoscopic exploration Laparoscopic Bariatric surgery Laparoscopic gynecological surgery	19 18 28 4 16 13	19.4 18.4 28.6 4.1 16.3 13.3	30 36 28 6 30 16	20.5 24.7 19.2 4.1 20.5 11.0	1.98 0.211 N.S.
**General abdominal surgery** No Appendectomy Caesarian Section Inguinal hernia Renal surgery	65 11 10 5 7	66.3 11.2 10.2 5.1 7.1	109 10 9 10 8	74.7 6.8 6.2 6.8 5.5	3.659 0.454 N.S.

(p) p value for comparing between the two studied groups; (*) Statistically significant at *p* ≤ 0.05

**Table 3:** Comparison between the two studied groups regarding the operative time

	Group I Umbilical trocar first “*n* = 98”	Group II Epigastric trocar first “*n* = 146”	Test *P* value
**Entry time (minutes)** Range Mean SD	6–11 8.6 1.9	4–5 4.5 0.5	4.22 0.001*
**Dissection time (minutes)** Range Mean SD	25–50 38.2 8.5	24–56 36.5 8.9	1.61 0.07
**Total operative time (minutes)** Range Mean SD	31–61 46.8 8.8	29–55 41.6 7.7	2.11 0.031*

(p) p value for comparing between the two studied groups; (*) Statistically significant at *p* ≤ 0.05

**Table 4:** Comparison between the two studied groups regarding intraoperative complications

Intra operative complications	Group I Umbilical trocar first “*n* = 98”		Group II Epigastric trocar first “*n* = 146”		*P* value T test
	No	%	No	%	
**G.B. perforation**	6	6.1	9	6.2	0.989
**CBD injury**	2	2.0	3	2.1	0.993
**Conversion to open laparotomy**	8	8.2	3	2.1	0.012*
**Colon injury**	1	1.0	1	0.7	0.775
**Small bowel injury**	6	6.1	1	0.7	0.006*
**Mesenteric injury**	4	4.1	0	0.0	0.065
**Vascular injury**	1	1.0	0	0.0	0.771
**Liver injury**	0	0.0	3	2.1	0.109
**Falciform injury**	0	0.0	3	2.1	0.526

(p) p value for comparing between the two studied groups; (*) Statistically significant at *p* ≤ 0.05

**Table 5:** Comparison between the two studied groups regarding the post operative data

Post-operative data	Group I Umbilical trocar first “*n* = 98”		Group II Epigastric trocar first “*n* = 146”		*P* value T test
	No	%	No	%	
**Incisional hernia**	1	1.0	2	1.4	0.808
**Postoperative jaundice**	1	1.0	1	0.7	0.775
**Wound infection**	2	2.0	3	2.1	0.993
**Abdominal collection**	1	1.0	2	1.4	0.808
**Pain score after 6 h** Range Mean SD	2–6 4.03 1.2		2–4 3.0 0.8		3.98 0.001*

(p) p value for comparing between the two studied groups; (*) Statistically significant at *p* ≤ 0.05

## Discussion

The choice of the optimal laparoscopic entry technique is still a topic of debate. Despite the overall safety of laparoscopic approaches, there is still a risk of serious unintended injuries to the intra-abdominal visceral structures occurring during the initial entry into the abdomen via the primary trocar [12, 13]. In order to reduce the risk of these complications, several entry maneuvers have been developed to lessen the likelihood of these complications with no universal recommendation exists regarding the safest approach [14, 15]. 

Previous reports indicate that the risk of intra-abdominal adhesions varies with surgical history. In their prospective single-centre study of 814 patients, Audebert and Gomel [16], reported periumbilical adhesions in up to 51.7% of patients with midline incisions. Another study by Ellis [17], reported that among 624 individuals with a history of abdominal surgery, intra-abdominal adhesions were present in 78% of the patients. Additionally, Pestieau, Marchettini [18], revealed that the likelihood of development of adhesions following abdominal procedures has been reported to be approximately 47% for appendectomies, rising to 91% in the case of pelvic surgeries.

The conventional location for the insertion of the Veress needle during peritoneal insufflation is at the umbilical base, as this area represents the thinnest portion of the abdominal wall. Nevertheless, this site may not be suitable for certain patients with history of laparotomy, ventral hernia, suspected intraperitoneal adhesions, or who have experienced unsuccessful access after three attempts. For these individuals, alternative entry points are available, with Palmars point being the most frequently suggested option [19]. 

This study compared LC using two laparoscopic entry techniques in patients with prior abdominal surgeries with resulting scar extending near the umbilicus with expected intraperitoneal adhesions; the traditional umbilical trocar-first approach using the Hasson open technique for entry and a novel epigastric trocar-first technique following pneumoperitoneum creation at Palmars point. Compared to the umbilical trocar-first group, patients in the epigastric trocar-first group had: Shorter entry time (4.5 ± 0.5 min vs. 8.6 ± 1.9 min, *p* = 0.001), lower small bowel injury rates (0.7% vs. 6.1%, *p* = 0.006(و reduced need for conversion to open surgery (2.1% vs. 8.2%, *p* = 0.012) and lower pain scores at 6 h postoperatively (*p* = 0.001).

The existence of adhesions resulting from prior surgical interventions has been observed to extend the duration of subsequent operations [20, 21]. Shakoor [22], reported that the average time required for access was 4.78 ± 1.43 min for the closed visual entry group, while the open entry group had an average of 6.11 ± 4.12 min. Similarly, Baruah [23], found that the mean access times were 5.62 ± 2.23 min for the closed entry technique using a Veress needle and 7.18 ± 2.52 min for the open method utilizing a Hasson cannula. In our study, the conventional open technique needed a significantly longer time for entry compared to our novel technique. This is attributed to the time taken in the open technique for layer by layer dissection of the abdominal wall at the incision site followed by the time taken for dissection of subsequent intraperitoneal adhesions. The reduction in the entry time with the epigastric trocar first technique may contribute to overall improved surgical efficacy and reduced operative stress.

Krishnakumar and Tambe [3], revealed that major organ damage has been documented at a frequency of 1.1 instances per 1000 laparoscopic access procedures. Of these, intestinal damages were observed at a rate of 0.7 per 1000, while significant vascular injuries were reported at a frequency of 0.4 per 1000. Minor complications, such as postoperative infection, subcutaneous emphysema, extraperitoneal insufflation, and trocar site hernia, are also associated with laparoscopic entry [24]. Our study revealed a significant increase in the incidence of small bowel injuries as well as conversion to open laparotomy among patients in the umbilical trocar first approach with the Hasson open technique used for entry. The increased safety in dissecting intraperitoneal umbilical adhesions under direct visualization with laparoscopic tools in the epigastric trocar first group is likely responsible for this outcome. In contrast, the incidence of injuries to the colon, vascular structures, and mesentery was comparable between the two groups in our study.

Pain at the time of discharge is a significant parameter for patient satisfaction. Virk [25], in their study comparing close (Veress Needle) versus Open (Hasson’s) entry Techniques for Creation of pneumoperitoneum in patients undergoing LC, found that 32(53.3%) and 33(55%) patients had no pain in close entry group and open entry group respectively at the time of their discharge from hospital. In our study, the umbilical trocar first group had a significantly more pain at 6th hour postoperatively compared with our novel technique.

These findings highlight that modifying the entry site for LC in high-risk patients due to previous abdominal surgeries had several clinical implications improving safety and surgical efficiency; enhanced Safety owing to reduced incidence of bowel injury by allowing early adhesiolysis under direct vision, lower conversion rates by minimizing entry-related complications and thus preventing unnecessary laparotomy, shorter operative times as a result of shorter entry time and lower postoperative pain due to less tissue trauma at the umbilicus which may contribute eventually to improved recovery.

Beyond laparoscopic cholecystectomy, this approach could be beneficial in other laparoscopic procedures where prior abdominal surgery increases the risk of adhesion-related complications. Potential applications include; laparoscopic hernia repair (to prevent adhesions near existing incisions), gynecological surgeries (for instance, hysterectomy in individuals with a history of previous cesarean deliveries), colorectal surgeries (for patients who have undergone previous midline incisions) and emergency laparoscopic procedures (where fast and secure access is essential). By incorporating this technique, surgeons with different specialties can tailor their approach based on patient history, improving overall safety in laparoscopy.

Additional multi-center trials with larger sample sizes and long-term follow-up are recommended to standardize this approach with additional studies needed to evaluate this approach in other laparoscopic procedures.

## Conclusion

Patients with previous abdominal surgeries present challenges in laparoscopic access due to potential adhesions at the umbilical trocar site. Our study demonstrates that an epigastric trocar-first approach, following pneumoperitoneum creation at Palmars point, significantly reduces the risk of small bowel injuries and conversion to open surgery compared to the traditional umbilical trocar-first method. This technique also results in shorter operative times and lower postoperative pain levels.

Given these findings, the epigastric trocar-first approach should be considered as a safer novel alternative for LC with difficult abdominal access owing to previous abdominal surgeries.

Beyond LC, this approach could be beneficial in other laparoscopic procedures where prior abdominal surgery increases the risk of adhesion-related complications. Potential applications could include colorectal surgery, gynecological procedures and emergency laparoscopic interventions, where rapid and secure abdominal access is required. Further multi-center studies and long-term follow-up are recommended to confirm its efficacy and explore its application in other laparoscopic procedures.

## Data Availability

No datasets were generated or analysed during the current study.
